# Genome-Wide Identification and Analyses of Calmodulins and Calmodulin-like Proteins in *Lotus japonicas*

**DOI:** 10.3389/fpls.2017.00482

**Published:** 2017-04-05

**Authors:** Jinqiu Liao, Jiabin Deng, Zongzhi Qin, Jiayong Tang, Maorong Shu, Chunbang Ding, Jing Liu, Chao Hu, Ming Yuan, Yan Huang, Ruiwu Yang, Yonghong Zhou

**Affiliations:** ^1^College of Life Sciences, Sichuan Agricultural UniversityYaan, China; ^2^School of Geography and Tourism, Guizhou Education UniversityGuiyang, China; ^3^Animal Nutrition Institute, Sichuan Agricultural UniversityChengdu, China; ^4^Triticeae Research Institute, Sichuan Agricultural UniversityChengdu, China

**Keywords:** *Lotus japonicus*, calmodulin, calmodulin-like protein, calcium, EF hands

## Abstract

*L. japonicus*, a model plant of legumes plants, is widely used in symbiotic nitrogen fixation. A large number of studies on it have been published based on the genetic, biochemical, structural studies. These results are secondhand reports that CaM is a key regulator during Rhizobial infection. In plants, there are multiple CaM genes encoding several CaM isoforms with only minor amino acid differences. Moreover, the regulation mechanism of this family of proteins during rhizobia infection is still unclear. In the current study, a family of genes encoding CaMs and CMLs that possess only the Ca^2+^-binding EF-hand motifs were analyzed. Using ML and BI tree based on amino acid sequence similarity, seven loci defined as *CaMs* and 19 *CMLs*, with at least 23% identity to CaM, were identified. The phylogenetics, gene structures, EF hand motif organization, and expression characteristics were evaluated. Seven CaM genes, encoding only 4 isoforms, were found in *L. japonicus*. According to qRT-PCR, four LjCaM isoforms are involved in different rhizobia infection stages. *LjCaM*1 might be involved in the early rhizobia infection epidermal cells stage. Furthermore, additional structural differences and expression behaviors indicated that LjCMLs may have different potential functions from LjCaMs.

## Introduction

A growing plant is forced to adapt to a variety of external stimuli due to the inconstant nature of its environment. Calcium (Ca^2+^) is a universal second messenger in plant signal transduction; it acts as a mediator in many processes associated with plant growth and development as well as the physiological responses to both abiotic (light, gravity, cold, heat, touch, wounding, drought, and oxidative stress) and biotic (phytohormones, pathogens, and symbiants) factors in plants (McCormack et al., 2005; Boonburapong and Buaboocha, 2007; Gifford et al., 2013; Abbas et al., 2014). To generate specificity in response, Ca^2+^ signals must be decoded by several Ca^2+^ sensors or Ca^2+^-binding proteins, which usually contain a number of paired EF-hand motifs and a helix-loop-helix structure (McCormack et al., 2005; Scholz et al., 2014). Currently, two major classes of Ca^2+^ sensors have been characterized in plants. One class is capable of transducing the signal via enzymatic activity and acts as a Ca^2+^ sensor or an effecter, such as CDPKs (Ca^2+^-dependent protein kinases), which contain a kinase domain and four EF-hands in a single protein. The other class contains non-catalytic proteins and acts only as Ca^2+^ sensors that upon Ca^2+^-induced conformational changes interact with and regulate many downstream target proteins. The primary groups of proteins found in this category include CBLs (calcineurin B-like proteins), CaM (Calmodulins), and CML (calmodulin-like proteins), which only contain EF-hand motif(s) (Yang and Poovaiah, 2003; Scholz et al., 2014).

CaM is a small multifunctional intermediate messenger protein and is one of the most conserved Ca^2+^-binding proteins in eukaryotes. It is typically comprised of 148 residues with four EF-hand motifs that upon binding to calcium ions change conformation. Each EF-hand motif contains two alpha helices that are connected by a 12 amino acid-residue loop (Yang and Poovaiah, 2003; Gifford et al., 2013). As is similar to what has been identified in animals, in which a single isoform of CaM is encoded by three separate genes (Fischer et al., 1988), multiple CaM genes encoding several CaM isoforms with only minor amino acid differences have been identified in several plants (McCormack and Braam, 2003; Boonburapong and Buaboocha, 2007; Gifford et al., 2013) including Arabidopsis (McCormack and Braam, 2003; Yang and Poovaiah, 2003; McCormack et al., 2005; Abbas et al., 2014), soybean (Sang et al., 1995; Heo et al., 1999; Gifford et al., 2013), pea (*Pisum sativum*; Oh and Roberts, 1990), petunia (*Petunia hybrid*; Rodriguez-Concepcion et al., 1999), rice (*Oryza sativa*; Phean-o-pas et al., 2005; Boonburapong and Buaboocha, 2007), tobacco (*Nicotiana tabacum*; Oh and Yun, 1999), and aloes leaf (*Aquilaria microcarpa*; Kurosaki and Taura, 2015). Evidence has accumulated supporting the theory that the presence of multiple diverged CaM isoforms may have distinct and significant functions, although gene redundancy cannot be ruled out as of yet. Particular stress signals, including both abiotic and biotic stimuli, have been reported to result in the differential expression of CaM isoforms in a single plant species. The differential Ca^2+^ sensitivity of CaMs may play an essential role in the selective target activation in different tissues in response to different stimuli by regulating CaM/target protein binding. For example, it has been shown that the expression levels of calmodulin isoforms in soybean, rice, and Arabidopsis are ubiquitous; however, they are differentially regulated by various stress signals (Heo et al., 1999; Phean-o-pas et al., 2005; Gifford et al., 2013; Abbas et al., 2014). Besides CaMs, plant tissues also have CMLs, which were found to share the same EF hand motifs with CaMs. The CMLs are thought to be involved in a variety of stress signals (Bender and Snedden, 2013). The *CML* genes have been reported to be induced by abiotic stresses (Vanderbeld and Snedden, 2007; Park et al., 2010; Vadassery et al., 2012). *CML*9, *CML*24 and *CML*37 have been reported to be induced by infection with the phytopathogenic bacterium *Pseudomonas syringae* and the herbivore *Spodoptera littoralis* (Delk et al., 2005; Leba et al., 2012; Scholz et al., 2014). *AtCML*24 has been found to play a role in seed germination and to act as a positive regulator of pollen germination and pollen tube development (Delk et al., 2005; Yang et al., 2014).

*L. japonicas*, a model plant of legumes plants, is widely used in symbiotic nitrogen fixation. During the symbiosis, calcium oscillation is thought to be decoded calcium/calmodulin-dependent protein kinase (CCaMK) which is characterized by the kinase domain, a calmodulin binding domain and the EF-hand domains (Gleason et al., 2006; Tirichine et al., 2006). In order to dissect the dual regulation of CCaMK by calcium, a large number of studies on the function of it in the symbiotic system have been published based on the genetic, biochemical, structural studies. These results are secondhand reports that CaM is a key regulator during Rhizobial infection (Liao et al., 2012; Shimoda et al., 2012; Miller et al., 2013). However, so far the calmodulin gene mutant plants in *L. japonicus* have not been found. There is also a lack of publications regarding the *CaM* gene family in *L. japonicus*. However, many questions are still unknown. For example, how many calmodulin genes in *L. japonicus*? During different rhizobial infection stages, which calmodulin family gene is expressed?

The progress being made in the whole genomic DNA sequencing project of *L. japonicus* enables the opportunity to isolate all of the sequences that encode CaMs and CMLs. A few interesting questions can begin to be addressed, such as how does these proteins exist in the genome of *L. japonicas*; and how many of the Lotus CaMs (LjCaMs) and CMLs (LjCMLs) are involved in nitrogen fixation symbiosis (NFS). In the present study, the genes encoding calmodulin and calmodulin-like proteins from the *L. japonicus* genome were identified. The insights that can be obtained from the identified characteristics including the EF-hand motif organization, the gene structures and expression, are also discussed. According to the Lotus gene expression atlas, *LjCML6, LjCML7*, and *LjCML12* are highly expressed in nodules, which may be involved in NFS. qRT-PCR was used to confirm the involvement of LjCaMs, LjCaM1, LjCaM2, LjCaM3, and LjCaM4 in NFS. These results lay the foundation for further study on the regulation mechanism of this family gene in nitrogen fixation symbiosis.

## Materials and methods

### Database searches and analyses of gene structures

The following three methods, HMMPfam, HMMSmat and superfamily, were used in order to search for Ca^2+^-binding proteins found in the Kazusa resource (http://www.kazusa.or.jp/lotus/) that do not possess functional domains other than the Ca^2+^-binding domain. Each method was used in order to identify proteins that have been shown to contain EF hand motifs or are in the family of Ca^2+^-binding proteins that contain the domains PF13499 and SM00054, and the protein family SSF47473. Additionally, BLAST searches (blastp) were carried out using the protein sequences of Arabidopsis CaM1 [AT5G37780] found in the Arapidopsis information resource (TAIR; http://www.arabidopsis.org) as the query sequences against the *L. japonicus* genome. Information was collected regarding the sequences information and each gene of interest. Using the loci identified in the Kazusa resource and performing comparisons between cDNA and genomic DNA sequences, searches were done in order to determine gene structures and locations.

### Construction of alignments and trees

All of the sequences used for constructing phylogenetic tree were untrimmed. The Maximum likelihood (ML) was analyzed in raxmlGUI1.3 (using default settings) with 1,000 replicates (Silvestro and Michalak, 2012). GTR + G was the calculation model. For Bayesian inference (BI), the matrix was carried out in MrBayes 3.1.2 (Huelsenbeck and Ronquist, 2001). We ran two separate analyses for 5 million generations each with Temp 0.10. Convergence and mixing were assessed using Tracer 1.6 (Suchard et al., 2001; Rambaut and Drummond, 2009). To evaluate convergence, we observed that the standard deviation of split frequencies fell below 0.01.

The LjCaMs were compared with those from other plant species using a multiple sequence alignment done using the ClustalX software. GenBank accession numbers for the sequences of other plant species used in the alignment include: *Saccharomyces cerevisiae* CaM CMD1p [GenBank: AAA34504], *Medicago truncatula* MtCaM1 [GenBank: xp_003624801]; *Oryza sativa* OsCaM1 [GenBank: NP_001049948]; *Glycine max* GmCaM1 [GenBank: NP_001238237].

### Determination of amino acid percent identity among proteins and motif analyses of proteins

The deduced amino acid sequences of the CaM and CaM-like proteins were aligned with LjCaM1 using ClustalX (Thompson et al., 1997). The percentage of identity between the pairs of proteins, the number of identical residues present throughout the alignment was calculated and summed and was then divided by the total number of amino acids that are present in the shorter protein sequences compared and was expressed as a percentage. This method was used to figure out the total percentage of identity between these two proteins. All the sequences were analyzed for the presence of EF-hand motifs and other functional domains using the InterProScan software (http://www.ebi.ac.uk/interpro/). The computer program: Myristoylator (http://web.expasy.org/myristoylator/) was used in order to locate protein sequences with the potential to be modified.

### Expressed sequence tags (ESTs)

The ESTs corresponding to *LjCaMs* and *LjCMLs* were identified by conducting BLAST search of the Lotus gene expression atlas (http://ljgea.noble.org/v2/). The expression characteristics of each gene were identified based on the types of library from which the ESTs were derived.

### 3′-race clone

Total RNA was extracted using the RNA Extraction Kit (TakaRa, Dalian, China). For RT-PCR, the first chain cDNA was synthesized using PrimeScript™ RT reagent Kit (TakaRa, Dalian, China) from nodule tissues of wild-type Gifu plants, and the random primer Oligo (dT) Primer was replaced by Oligo (dT) -3′-site primer. Using this cDNA as template, the *LjCaM* sequences which contain complete open reading frame (ORF) and polyA were amplified by forward primers (Table 1) and 3′-site adapter primer. The PCR conditions used were as follows: 94°C for 5 min, followed by 30 cycles of 94°C for 30 s, 58°C for 30 s, and 72°C for 50 s, with a final extension of 10 min at 72°C. The target fragments were purified and ligated with pMD19-T vector. The positive clones were sequenced by Sangon Biotech (Shanghai, China).

**Table 1:** **The sequences of the primers which were used in this study**.

**Gene**	**Primer sequence (from 5' to 3')**
**3'-RACE CLONING**	
*LjCaM1*	F: AGATCATTTTCCTCTCCATTCCA
*LjCaM2*	F: ATCCAATCCTATCCTATCACATC
*LjCaM3*	F: ACAACAATGGCAGATGTTCTGAG
*LjCaM4*	F: TCCAATGACAGATATCTTGAGTG
**Q-PCR ANALYSE**	
*Ubiquitin*	F: TGGTTTATTTGGGCCTTTTATGG R: GGCCAGAAGAGGCCACAAC
*NIN*	F: GATTGCTGTTGGGTACTTGAAAGAC R: AAGGGCACCCATATCTGAATGA
*LjCaM1*	F: AGGTCATGATGGCCAAGTGATC R: CCTAACTATATTTCTTTGTCTCTCCTTTTGTT
*LjCaM2*	F: TCGTTAAGATGATGATTACCATTGGAT R: TCTTGCAGTTGAATGAGACGATTAA
*LjCaM3*	F: TTCCACAACACACACATCACAAGA R: GGTGAGCTGATCGGCCATT
*LjCaM4*	F: AGATGATGATGACTGTTCGATGAAAC R: CAAGAACACAATATTTCATGGGAGG

### Q-PCR analyses of mRNA abundance

Seeds of wild-type Gifu B-129 plants were surface-sterilized and grown on wet filter paper for 7 days and then transplanted into vermiculite mixed soil (vermiculite:perlite = 3:1; Liao et al., 2012). These plants were separated into group A and B. The roots of plants in group A were collected after transplanted 9 days. This sample was the uninoculted roots. *Mesorhizobium loti* strain NZP2355 was inoculated with seedlings in group B 1 week after transplanting. After inoculated 2, 7, 14, and 21 days, the roots and nodules were collected, respectively.

Total RNA was isolated from different *L. japonicus* roots and nodules using an RNA Extraction Kit (TakaRa, Dalian, China). Using NanoDrop 1000 (Thermo, USA), the concentration and purity of RNA were determined by measuring absorbance at 260/280 nm. cDNA was prepared from 2 μg of total RNA using PrimeScript™ RT reagent Kit (TakaRa, Dalian, China). Q-PCR was performed in triplicate (i.e., three biological and three technical replicates) on the CFX-96 Real-Time PCR Detection System (*Bio-Rad*, USA) using SYBR Premix Ex Taq™ (TakaRa, Dalian, China). The reference gene, *Ubiquitin*, was used to normalize the results. The *NIN* gene was used as a positive control to demonstrate that the root nodule symbiotic model was successfully constructed. The primers (Table 1) for the 2 reference genes and 4 *LjCaM* genes were designed using Primer Express 3.0 (Applied 156 Biosystems, Foster City, CA).

For the analysis, ΔΔCt was used. The difference of samples should be normalized by reference genes *Ubiquitin* (ΔCt = Ct_targ._-Ct_norm_), followed by comparison of treated and control samples (ΔΔCt = ΔCt_treated_- ΔCt_control_). Finally, the expression was calculated by formula 2^−ΔΔCt^. The graphs were made by Excel and Adobe Illustrator CS3.

## Results

### Identification of EF-hand containing proteins in *L. japonicus*

First, in order to identify EF-hand-containing proteins, the *L. japonicus* genome available in the Kazusa Resources for Interpro Database Matches was searched using three different methods, HMMPfam, HMMSmart and superfamily. Second, the *L. japonicus* database was searched with AtCaM1 as the query amino acid sequence using the program BLASTp, and the unfound five sequences (*E* > 3e-64) were added to the list. Each identified amino acid sequence was analyzed for EF hands motifs and other functional domains using the InterProScan software using the default settings. A total of 47 putative proteins were identified that do not contain any other identifiable domains other than the Ca^2+^-binding domain in Lotus. The amino acids in the identified proteins are 23% identical with LjCaM1 and were analyzed further (Table 2).

**Table 2:** **Characteristics of ***LjCaM*** and ***LjCML*** genes and the encoded proteins**.

**Name**	**Locus^a^**	**Chr^b^**	**cDNA length^c^**	**Amino acids^d^**	**EF hands^e^**	**% of Met^f^**	**Identity to LjCaM1(%)^g^**	**Cys 27^h^**	**Lys 116^i^**	**Myristoylation^j^**
*LjCaM1-1*	Lj0g3v0233429.1		450	149	4	6.04	100	+	+	−
*LjCaM1-2*	Lj0g3v0233429.2		450	149	4	6.04	100	+	+	−
*LjCaM1-3*	Lj0g3v0233429.3		450	149	4	6.04	100	+	+	−
*LjCaM2*	Lj1g3v4047180.1	1	450	149	4	6.04	98.66	-	+	−
*LjCaM3-1*	Lj0g3v0048629.1		453	150	4	5.33	79.19	+	+	−
*LjCaM3-2*	Lj6g3v0001230.1	6	453	150	4	5.33	79.19	+	+	−
*LjCaM4*	Lj5g3v1208190.1	5	453	150	4	5.33	79.19	+	+	−
*LjCML1*	Lj6g3v0434040.1	6	420	139	4	6.47	42.45	−	+	−
*LjCML2*	Lj6g3v0874290.1	6	477	158	4	5.70	40.28	−	+	−
*LjCML3*	Lj2g3v2571120.1	2	423	140	4	5.00	40.00	−	+	−
*LjCML4*	Lj6g3v0874290.2	6	654	217	4	5.99	39.86	−	+	−
*LjCML5*	Lj6g3v0874290.3	6	564	187	4	6.42	39.86	−	+	−
*LjCML6*	Lj0g3v0192369.1		600	199	3	5.53	38.16	−	−	−
*LjCML7*	Lj2g3v0025910.1	2	336	111	2	4.50	35.71	−	−	−
*LjCML8*	Lj0g3v0335859.1		417	138	4	5.07	35.11	−	−	−
*LjCML9*	Lj3g3v2515300.1	3	420	139	3	7.19	34.56	−	−	−
*LjCML10*	Lj4g3v1388360.1	4	423	140	3	7.14	34.31	−	−	−
*LjCML11*	Lj1g3v2560490.1	1	354	117	2	4.27	32.10	−	−	−
*LjCML12*	Lj4g3v0912460.1	4	348	115	2	7.83	31.58	−	−	−
*LjCML13*	Lj1g3v4263370.2	1	714	237	3	3.80	31.51	−	−	−
*LjCML14*	Lj1g3v2035110.1	1	564	187	1	5.35	30.67	−	−	+
*LjCML15*	Lj0g3v0250669.1		444	147	3	7.48	30.61	−	−	−
*LjCML16*	Lj1g3v2560500.1	1	546	181	2	4.97	30.20	−	−	−
*LjCML17*	Lj3g3v0766150.1	3	441	146	3	8.90	28.17	−	−	−
*LjCML18*	Lj5g3v0890570.1	5	405	134	3	3.73	27.27	−	−	+
*LjCML19*	Lj1g3v3329980.1	1	519	172	1	3.49	23.91	−	−	−

a *Gene ID were downloaded from Kazusa Resources*.

b Chromosome numbers in which the gene resides

c *Length of the coding region in base pairs*.

d *Number of amino acids of the deduced amino acid sequence*.

e *Number of EF hands based on the prediction by interProScan*.

f *Percentage of methionine (M) residues in the deduced amino acid sequence*.

g *Number of identical residues divided by the total number of amino acids that have been aligned expressed in percentage*.

h *Presence of a cysteine equivalent to Cys27 of typical plant CaMs at residue 7 (−Y) of the first EF-hand*.

i *Presence of a lysine equivalent to Lys116 of typical plant CaMs*.

j *Presence of a putative myristoylation site*.

### Phylogenetic and protein sequence analysis of the lotus EF-hand family

**Figure 1:**
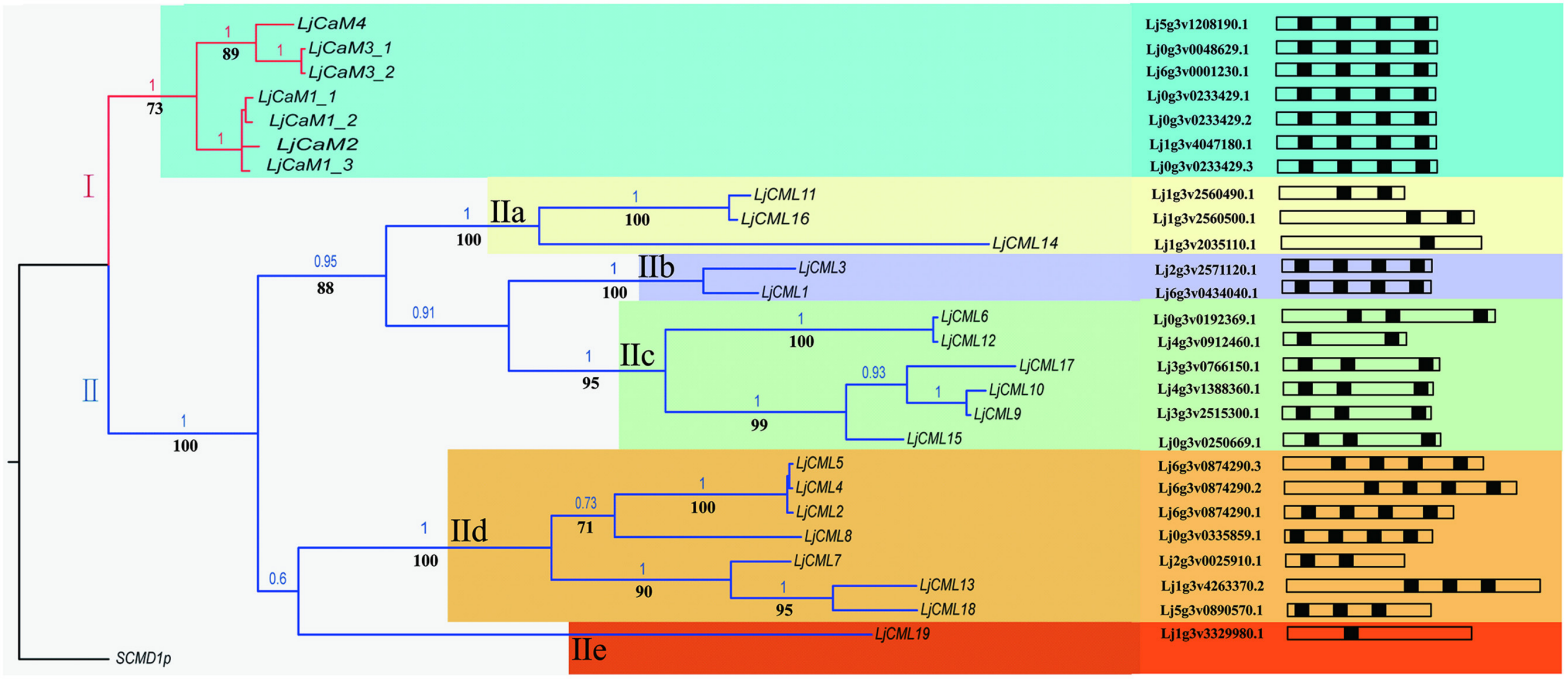
**Phylogenetic tree based on amino acid similarities among LjCaM and LjCML proteins**. Bayesian inference (BI) tree separates six groups of CaMs and CMLs, as indicated by colors. Non-significant bootstrap support falling below 70% and posterior probabilities <90% were not included. Posterior probabilities (PP) above and bootstrap support (BS) below the branches. Schematic diagrams of the LjCaM and LjCML open reading frames show their EF hand motif distribution.

**Figure 2:**
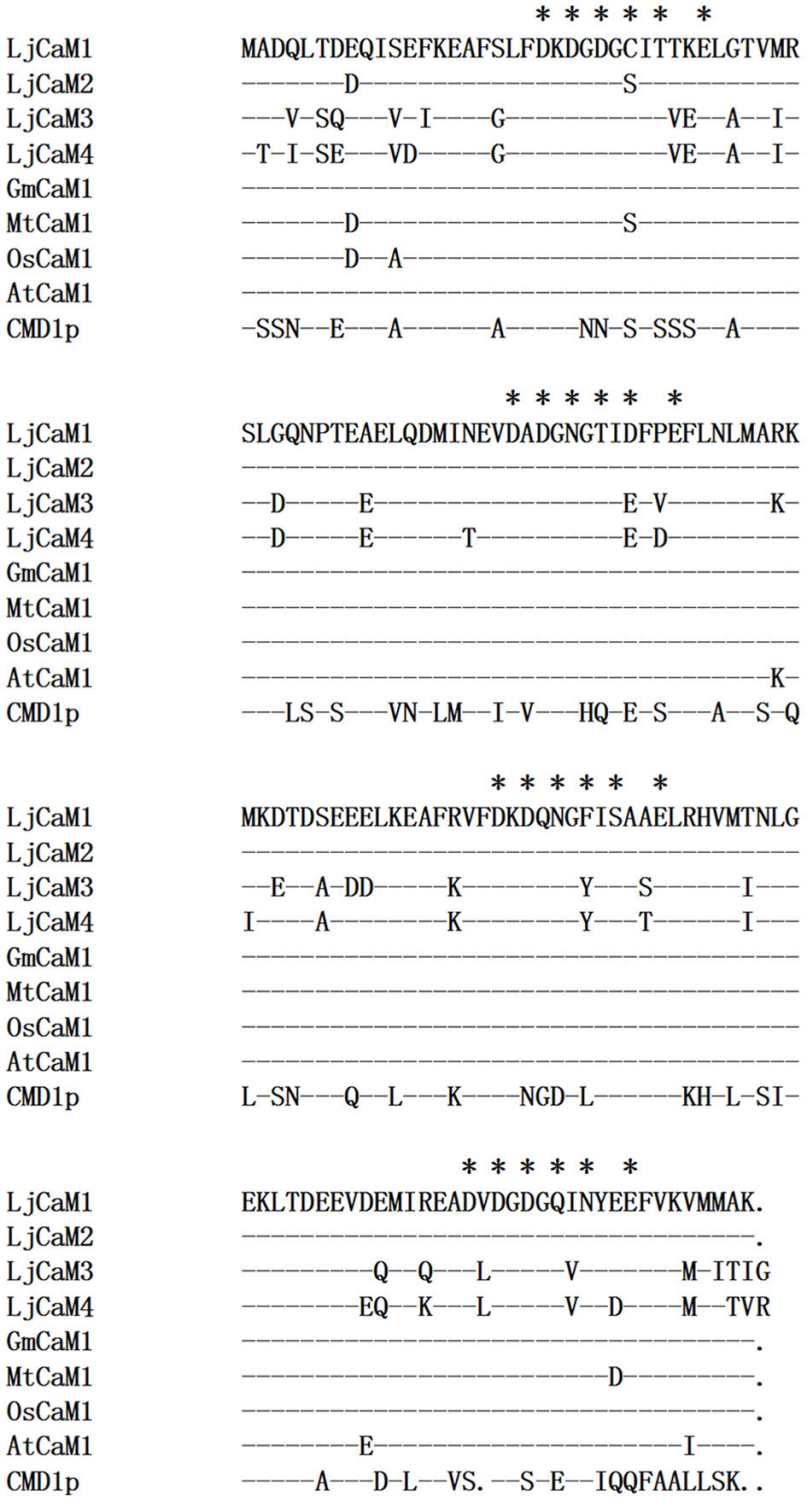
**LjCaM protein sequence similarity with CaM from other species**. Comparison of the deduced amino acid sequences of LjCaM1, LjCaM2, LjCaM3, and LjCaM4 with those of other plants and *Saccharomyces cerevisiae* CaM (CMD1p). The sequences are compared with LjCaM1, which was used as a standard; identical residues in other sequences are indicated by a dash (-), and a gap introduced for alignment purposes is indicated by a dot (.). Residues that serve as Ca^2+^-binding ligands are marked with asterisks (^*^).

ML and BI analyses produced major rule consensus trees with identical topologies except for the different BI posterior probability values and ML bootstrap values. The BI tree illustrated in Figure 1 shows posterior probabilities (PP) above and bootstrap support (BS) below the branches. Non-significant bootstrap support falling below 55% and posterior probabilities <75% were not included in Figure 1. The proteins were separated into two groups. Group I consisted of seven proteins and a multiple sequence alignment was performed with these proteins and known CaMs of other species, the results of which are shown in Figure 2 and depict their high-degree of amino acid identity (≥98%). *LjCaM1-1, LjCaM1-2*, and *LjCaM1-3* encode identical proteins called LjCaM1, whereas *LjCaM*2 encodes a protein with 2 amino acids difference from LjCaM1, and shares 98.66% identity with LjCaM1 (Table 2, Figure 2). *LjCaM*3-1 and *LjCaM*3-2 encode identical proteins, called LjCaM3, with only 19 amino acids difference from *LjCaM*4 (Table 2, Figure 2). LjCaM3 and LjCaM4 proteins share more than 79% identity with LjCaM1 (Table 2). The multiple sequence alignment of the LjCaM amino acid sequences with the known CaMs of other species, shown in Figure 2, shows a high degree of sequence conservation. It should be mentioned that the amino acid sequences of LjCaM1 are identical to the typical CaMs from Soybean (*G. max*) and Alfalfa (*M. truncatula*), which reflects the close relationships that exist among dicotyledon.

LjCaMs are methionine (M)-rich proteins (Table 2); with approximately 6.04% of their amino acids being methionine. Methionine residues contribute to almost half of the accessible surface area of the hydrophobic patches of CaM proteins, which allows for the interaction of these proteins with target proteins in a sequence-independent manner (O'Neil and DeGrado, 1990). Upon Ca^2+^-binding, CaM undergoes structural alterations which results in the exposure of the hydrophobic regions. The hydrophobic residues that contribute to the hydrophobic interactions are highly conserved and are critical in mediating formation of CaM-target protein complex, which is known to be critical for CaM function. Additionally, the lysine^116^ (K) is present in all LjCaM proteins and is assumed to be conserved for functional reason (Figure 2). Trimethylated Lysine^116^ is believed to be a posttranslational modification that aids in the regulation of CaM activity. Thus, these CaMs may function like typical CaMs and were classified as “true” CaMs. Their characteristics are summarized in Table 2.

The rest of the proteins from phylogenetic tree as shown in Figure 1 formed group II. The remaining proteins were called LjCMLs based on their percentage of amino acid identity with LjCaM1, which was calculated as described above. The LjCMLs are predicted to be relatively small proteins, being comprised of 111–237 amino acids, and were shown to have an amino acid identity between 23.91 and 42.45% with LjCaM1. According to the phylogenetic tree, LjCML proteins were separated into five groups: a, b, c, d, and e (Figure 1). The LjCML proteins in IIa, IIb, IIc, IId, and IIe have an average of 30.99, 41.23, 32.90, 35.66, and 23.91% identity with LjCaM1, respectively.

Similar to what is found in LjCaMs (in group I), 19 of the *LjCML* genes are composed of EF hands without other functional domains (Table 2, Figure 2). The number of EF hands present in the *L. japonicus* CML proteins ranged from 1 to 4. LjCML14 and LjCML19 each only have one EF hand. The majority of LjCMLs have either two pairs, or one pair, of identifiable EF hands with the exception of LjCML12, which appears to have two separate EF hand motifs. A total of 7 LjCML proteins, LjCML6, LjCML9, LjCML10, LjCML13, LjCML15, LjCML17, and LjCML18, which have one pair of EF hands, have an extra EF hand motif that does not pair with any other motifs (Figure 1). The pairing of EF-hand motifs in the CaM protein increases its affinity for Ca^2+^, therefore an unpaired EF hand in these proteins may bind to Ca^2+^ with a lower affinity, or may be non-functional.

The amino acid at position 27 in the first EF hand, which is assumed to be a cysteine (C), is absent in all LjCMLs (Table 2). Based on the multiple sequence alignment, LjCML1, LjCML2, LjCML3, LjCML4, and LjCML5 contain a lysine^116^, which is the same as what is found in the CaMs. A high percentage of methionine (M) residues, which is another important determinant of CaM function, has also been observed in most of LjCMLs. The average percentage of methionine in the LjCML proteins is 5.73%. The Met-rich feature found in LjCMLs is considered to be an indication of their relatedness to CaMs because of the low percentage of M residues typically found in other proteins. Therefore, CMLs may have similar mechanisms of action with CaMs, such as the exposure of hydrophobic residues as a result of conformational changes that occur upon Ca^2+^ binding. Moreover, LjCML21 only has a methionine content of 3.49%, which suggests that its mode of action upon Ca^2+^ binding is probably different from the hydrophobic surface exposure upon the conformational changes of CaM. Despite this, some characteristics specific to CMLs are most likely fine-tuned to their Ca^2+^-regulated activity associated with plant growth and development as well as the physiological responses to both abiotic and biotic factors in plants.

Potential modification sites present in the amino acid sequences were determined using the computer program Myristoylator. The results indicated that LjCML14 and LjCML18 contain a potential myristoylation sequence (Table 2).

### Characteristics of EF hands in *L. japonicus* proteins

**Figure 3:**
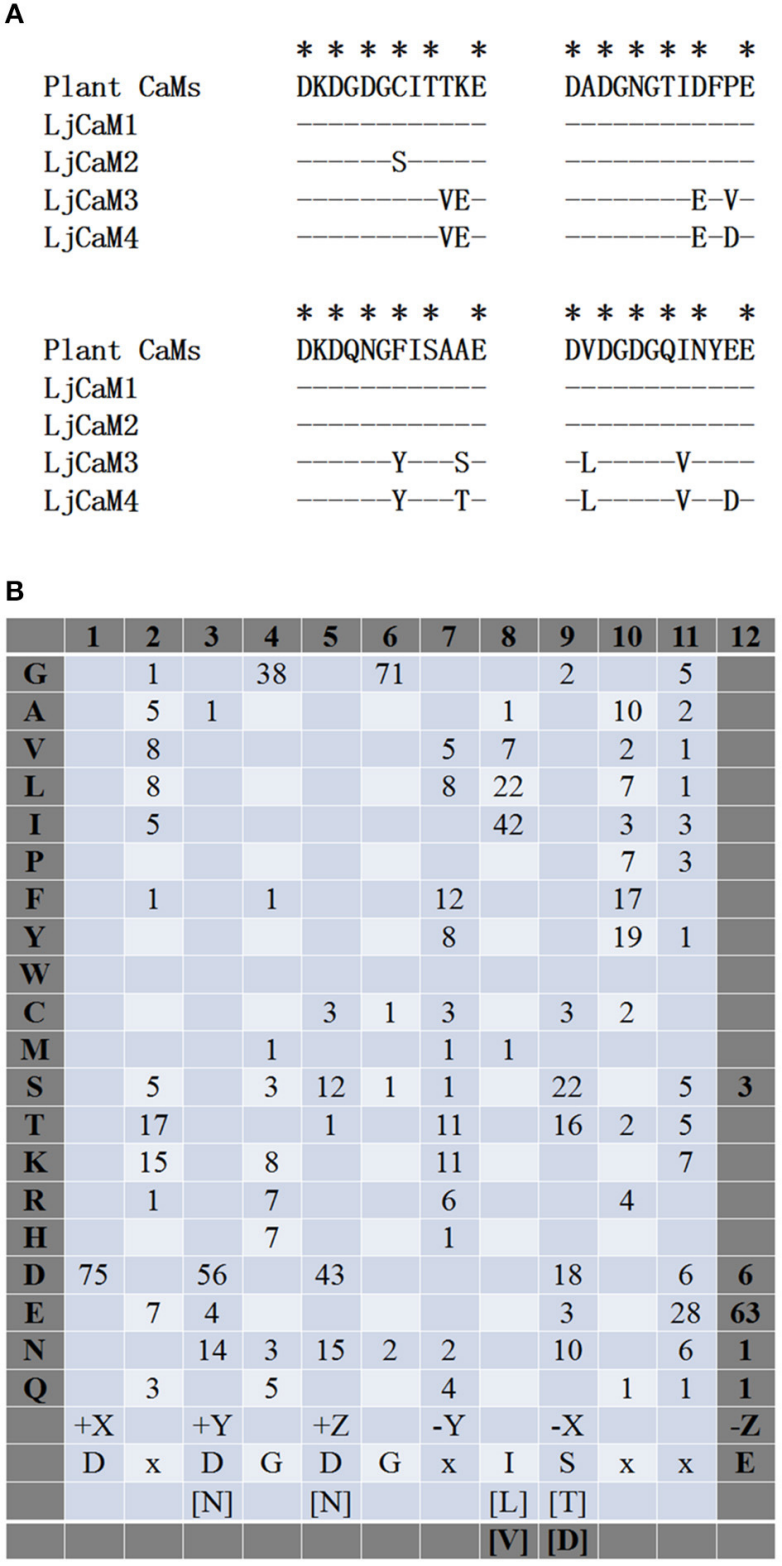
**Characteristics of EF hands in ***L. japonicus*** proteins. (A)** Residues in EF hands #1-4 of the LjCaMs are compared with the typical plant CaMs and the *S. cerevisiae* CaM (CMD1p) using a consensus sequence of plant CaMs as a standard; identical residues in other sequences are indicated by a dash (-). **(B)** Residues in Ca^2+^-binding loops in the 19 LjCML proteins are shown as the frequency at which an amino acid (shown at the left) is found at each position (shown at the top). The amino acids found most frequently are indicated with bold letters and are shown below as a consensus sequence along with the positions of residues that serve as Ca^2+^-binding ligands, which are indicated in Cartesian coordinates. Bracketed residues are alternative residues that are found frequently in each position and “x” is representative of a variety of amino acids.

Four LjCaM proteins with two pairs of EF hands were found, which was found in plant CaMs. The comparison results of the sequence of the Ca^2+^-binding site in the EF hands between plant CaMs and LjCaM1, LjCaM2, LjCaM3, and LjCaM4 are shown in Figure 3A. The Ca^2+^-coordinating residues of LjCaM1 are the same as those of the plant CaM. This hallmark of higher plant CaM sequence is absent in LjCaM2, which has a Serine (S^26^) at residue 7 (−Y) of the first EF hand instead. Residue 7 (−Y) acts through its main-chain oxygen, which is different from the other residues that interact with Ca^2+^ through side-chain oxygens (McCormack and Braam, 2003). The residues in positions 1, 3, 5, 9 and 12 (alternatively called +X, +Y, +Z, −X, and −Z in Figure 3A) of the Ca^2+^-binding loop are also conserved among the LjCaMs. The residue at position 1 (+X) in the 16 EF hand motifs of the LjCaMs is exclusively occupied with an aspartate (D); the residue at position 3 (+Y) is typically an aspartate (D); the residue at position 5 (+Z) is usually an aspartate (D) or an asparagine (N); and the residue at position 9 (−X) is either threonine (T), aspartate (D), serine (S), or asparagine (N), which are all commonly found in plant CaMs; and the residue at position 12 (−Z) is glutamate (E), which is invariable and found at this position in most Ca^2+^-binding EF hand motifs. This residue may rotate in order to coordinate bidentate or monodentate metal ion chelation. Glutamate provides two sites of coordination that favor Ca^2+^ over Mg^2+^ coordination.

A few important CaM functions were found to exist in only a subset of CaM-like proteins, although, based on the phylogenetic tree, many family members appear to be a big distance from the LjCaMs. The amino acids comprising the 55 Ca^2+^-binding loops in the 19 CML proteins were aligned and the percentages of specific amino acids are shown in Figure 3B. The ligands for Ca^2+^ coordination in the EF-hand motifs of LjCMLs are highly conserved, which suggests that most of them are functional EF hands. As is seen in CaMs, the residue at position 1 (+X) is exclusively an aspartate (D); and the residue at position 3 (+Y) and 5 (+Z) are typically an aspartate (D) or an asparagine (N). The glycine (G) at position 6 is conserved in each of the 55 EF hands in the LjCML proteins. However, a subset of individual CMLs was shown to have significant sequence divergence in the Ca^2+^-binding loops. The majority of the residues in the Ca^2+^-binding loops are conserved among the LjCML proteins, the residue at position 12 (−Z) is primarily glutamate (E), with the exception of an EF hand in LjCML6, LjCML9, LjCML10, LjCML12, LjCML15, and LjCML17, in which there is an aspartate (D) at this position. LjCML6, LjCML9, LjCML10, LjCML15, and LjCML17 possess one separate EF hand and one pair of EF hands with an aspartate at residue 12 in the EF hand motif at the carboxyl terminus, whereas the LjCML12 has two separate EF hand motifs. An aspartate (D) substitution for a glutamate (E) at the 12^th^ position of the Ca^2+^-binding loop, results in increased binding affinity of EF hands for Mg^2+^ (Houdusse and Cohen, 1996; Cates et al., 2002).

### *LjCaMs* and *LjCMLs* gene structures

**Figure 4:**
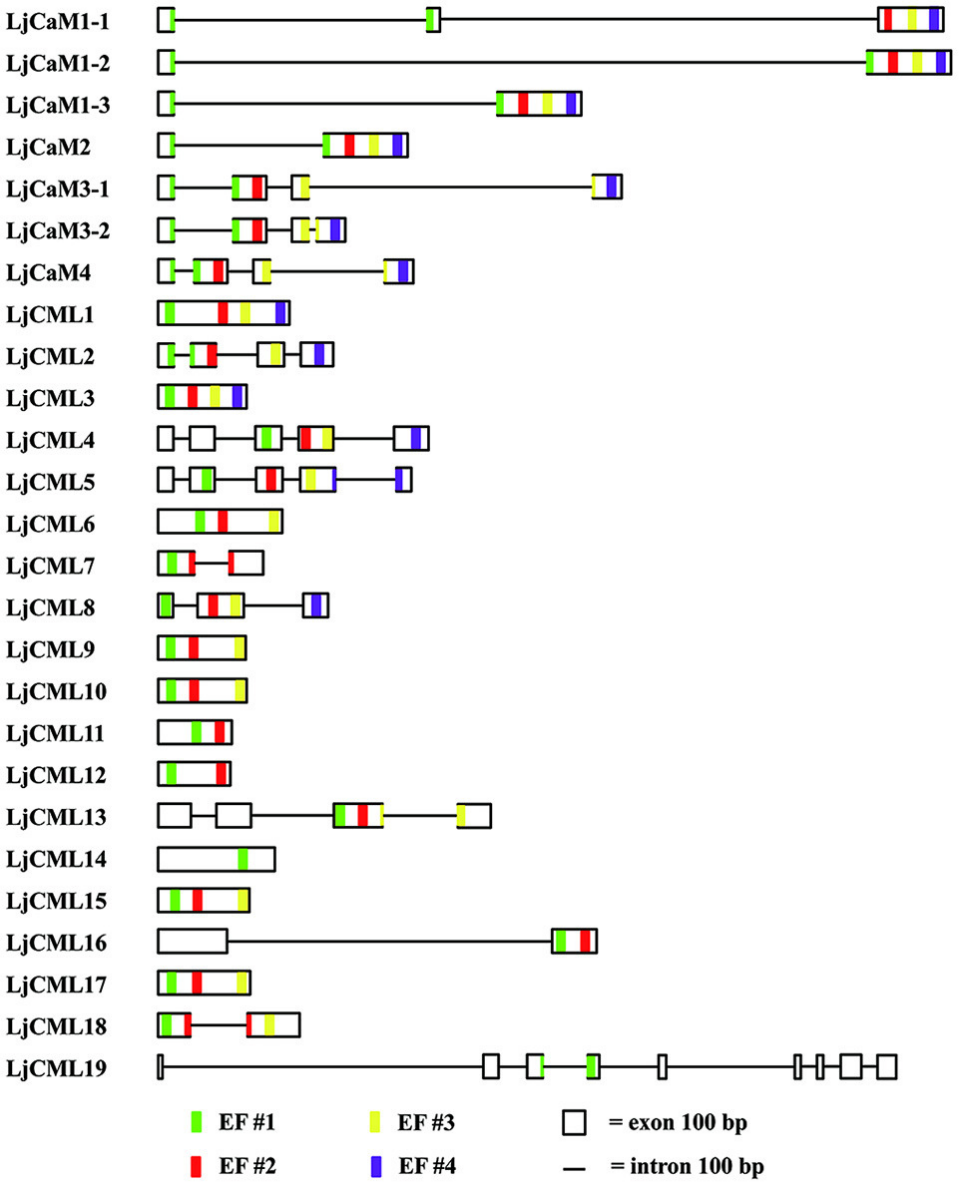
**Predicted presence and prediction of introns, exons and EF hand-coding sequences in ***L. japonicus*** CaMs and CMLs**. Intron and exon boundaries were determined using comparisons of genomic DNA with cDNAs or were predicted based on the genomic sequences. EF hands were identified by the presence of canonical sequences (see Figure 2, 3B) and an alignment with related CaMs and CMLs (Figure 1), as described in the text. Thin lines represent introns, boxes represent exons and EF-hand motif #1, #2, #3, and #4 are represented by green, red, yellow and pink stripes at their positions, respectively. The size marker at bottom indicates a distance of 100 bases.

The structures of *LjCaMs* and *LjCMLs* were determined through a comparison of their cDNAs with the corresponding genomic DNAs. The schematic diagrams are shown in Figure 4. Out of 26 *LjCaM* and *LjCML* genes, 16 of the genes contain intron(s); none of them are present in groups IIb or IIc (Figure 1). It should be noted that the *LjCaM1-1, LjCaM1-2*, and *LjCaM1-3* were identified to have an alternatively spliced mRNA that encodes LjCaM1. *LjCaM1-1* has an additional intron compared with *LjC*aM1-2, *LjCaM1-3*, and *LjCaM2* (Figure 4). The *LjCaM3-1, LjCaM3-2*, and *LjCaM4* genes have the same gene structure, containing 3 introns. *LjCaM3-1* and *LjCaM3-2* were shown to encode the same protein, LjCaM3. Despite the presence of nucleotide variability, the multiple genes encode the same proteins suggesting selective pressure to strictly maintain the amino acid sequence. Therefore, the *L. japonicus* CaMs show a similar sequence conservation. This sequence conservation could be an example of genomic redundancy; however, it is difficult to explain how natural selection acted in order to maintain identical protein sequences. If the multiple *CaM* genes are truly redundant, one would expect some sequence divergence at least among the genes from distinct species (Toutenhoofd and Strehler, 2000). One possibility is that the *CaM* genes are differentially expressed and therefore the products function with spatial or temporal specificity (Toutenhoofd and Strehler, 2000). Interestingly, all of the *LjCaM* genes (Figure 1) are interrupted by an intron at their coding regions within the Gly^26^. *LjCML2, LjCML4*, and *LjCML5*, which have different gene structures, encode a slightly different protein (Table 2, Figure 4).

### Phylogenetic analysis of *L. japonicus* and other species CaMs

**Figure 5:**
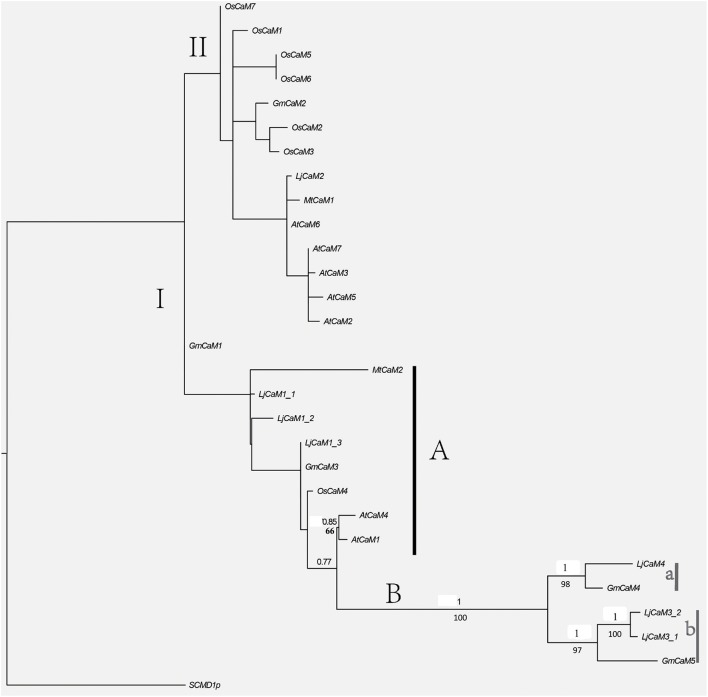
**Phylogenetic relationships among ***L. japonicus*** and other species CaM proteins**. BI and ML analyses produced major rule consensus trees with identical topologies except for the different BI posterior probability values and ML bootstrap values. The ML was analyzed in raxmlGUI1.3 (using default settings) with 1,000 replicates. I+GTR was as the calculating model. The BI tree was employed in MrBayes 3.2 with default settings to access the relationship within the gene family. Two separate analyses for 400,000 generations with 25% burned were performed. Non-significant bootstrap support falling below 55% and posterior probabilities <75% were not included. Posterior probabilities (PP) above and bootstrap support (BS) below the branches. GenBank accession numbers for the sequences of other plant species used in the alignment were from *M. truncatula MtCaM*2 [GenBank: XP_003618875.1], Sang et al. (1995), McCormack and Braam (2003) and Boonburapong and Buaboocha (2007).

Alignment of LjCaMs with that of other species were subjected to phylogenetic analysis (Figure 5). Several LjCaMs were shown to have high levels of similarity with those of other species. Most of the CaMs and LjCaMs proteins are highly conserved, with approximately 93.50–97.99% identity. Interestingly, all of the calmodulin proteins were separated into two groups I and II. *LjCaM*2 nested in group II has a very significant divergence with other *LjCaMs*. In group I, there are subgroup A and B. *LjCaM*1*-*1, *LjCaM*1*-*2, and *LjCaM*1*-*3 are also differentiation from *LjCaM3-*1, *LjCaM3-*2, and *LjCaM4*. *LjCaM3-*1, *LjCaM3-*2, and *GmCaM*5 were grouped together with a high posterior probabilities in group b. And *LjCaM4* and *GmCaM*4 were also claded in group a. These five genes nested in subgroup B, which is significantly separated from the other calmodulin genes. *GmCaM*4 and *GmCaM*5 have an amino acid identity of 78.67% with *GmCaM*3. And *GmCaM*4 has an amino acid identity of 87.33% with *GmCaM*5. The similar situation in *L. japonicus* also exists (Table 2).

### *LjCaMs* and *LjCMLs* expression

The identification of expressed sequence tags (ESTs) corresponding to the *LjCaM/LjCML* can provide more information for *LjCaM/LjCML* expression (Table 3). All the *LjCaM* genes are highly expressed in nodule. And there were significant differences in the expression of these genes at different rhizobal infection stages. The *LjCML*s cluster into five major groups (Figure 1). Group IIa and IIb, comprising three genes and two genes respectively, have the highest expression at flowering stage. The third gene cluster (IIc) has the highest levels of expression during nodule developmental stages. Expression from the next seven genes peaks when noudle formation begins. The last group IIe has enhanced expression during seed development stage (Figure 1, Table 3). It is suggested that these *LjCMLs* might be involved in the molecular regulation of different development stages. A few *LjCML*s are highly expressed in specific organs such as *LjCML1, LjCML11*, and *LjCML16* in flowers, *LjCML6, LjCML7*, and *LjCML12* in nodules, *LjCML14* in roots, and *LjCML19* in seeds. This suggests that they are genes with a variety of different potential biological functions. In addition, the expression of *LjCML1, LjCML3, LjCML6, LjCML7, LjCML12*, and *LjCML14* under biotic stress (bacteria) is significantly different.

**Table 3:** **The ***LjCaM***s and ***LjCML***s gene expression atlas in organ-specific and bacteria induced**.

**Gene name**	**Seeds**	**Flower**	**Leaf**	**Stem**	**Root**	**Nodule**	**R3W Un^a^**	**R3W In7^b^**	**R3W In21^c^**
*LjCaM1-1*	3598.2	3969.61	3041.73	3661.08	2940.14	9322.85	2491.62	4373.77	4000.25
*LjCaM1-2*	3598.2	3969.61	3041.73	3661.08	2940.14	9322.85	2491.62	4373.77	4000.25
*LjCaM1-3*	3598.2	3969.61	3041.73	3661.08	2940.14	9322.85	2491.62	4373.77	4000.25
*LjCaM2*	3598.2	3969.61	3041.73	3661.08	2940.14	9322.85	2491.62	4373.77	4000.25
*LjCaM3-1*	317.61	27.37	35.53	595.56	749.8	2617.01	299.02	474.29	543.74
*LjCaM3-2*	317.61	27.37	35.53	595.56	749.8	2617.01	299.02	474.29	543.74
*LjCaM4*	317.61	27.37	35.53	595.56	749.8	2617.01	299.02	474.29	543.74
*LjCML1*	712.85	1373.57	934.38	946.28	276.82	174.27	571.21	236.49	829.42
*LjCML2*	79.4	181.68	122.35	402.88	467.25	358.14	260.37	208.24	260.17
*LjCML3*	137.81	835.28	172.57	337.04	593.82	222.81	1001.67	469.99	1348.77
*LjCML4*	79.4	181.68	122.35	402.88	467.25	358.14	260.37	208.24	260.17
*LjCML5*	79.4	181.68	122.35	402.88	467.25	358.14	260.37	208.24	260.17
*LjCML6*	296.31	229.89	57.11	571.28	653.1	2203.14	436.67	856.92	702.97
*LjCML7*	247.68	563.04	616.6	551.49	597.16	1546.73	441.23	1170.35	521.81
*LjCML8*	14.53	14.44	11.41	11.77	18.22	12.7	15.86	14.79	13.7
*LjCML9*	28.7	24.2	25.06	37.74	26.26	24.93	34.82	31.05	54.5
*LjCML10*	28.7	24.2	25.06	37.74	26.26	24.93	34.82	31.05	54.5
*LjCML11*	11.71	1643.78	12.83	54.74	1009.61	589.54	669.34	617.9	853.71
*LjCML12*	296.31	229.89	57.11	571.28	653.1	2203.14	436.67	856.92	702.97
*LjCML13*	156.66	180.86	137.16	259.04	350.5	117.8	367.95	288.23	381.15
*LjCML14*	28.38	28.81	29.2	45.01	1124.8	313.48	1046.65	476.64	761.15
*LjCML15*	28.7	24.2	25.06	37.74	26.26	24.93	34.82	31.05	54.5
*LjCML16*	11.71	1643.78	12.83	54.74	1009.61	589.54	669.34	617.9	853.71
*LjCML17*	14.12	10.07	9.68	36.06	10.81	12.82	24.37	19.56	104.25
*LjCML18*	605.28	419.78	88.52	146.93	199.36	143.35	172.24	161.62	143.54
*LjCML19*	1485.3	1046.29	685.67	572.63	664.97	1293.65	429.36	441.95	442.5

a *Wild type Gifu 3 weeks uninoculated roots*.

b *Wild type Gifu 7 days after inoculated root+nodule*.

c *Wild type Gifu 21 days after inoculated root+nodule*.

### mRNA abundances of *LjCaM* genes

**Figure 6:**
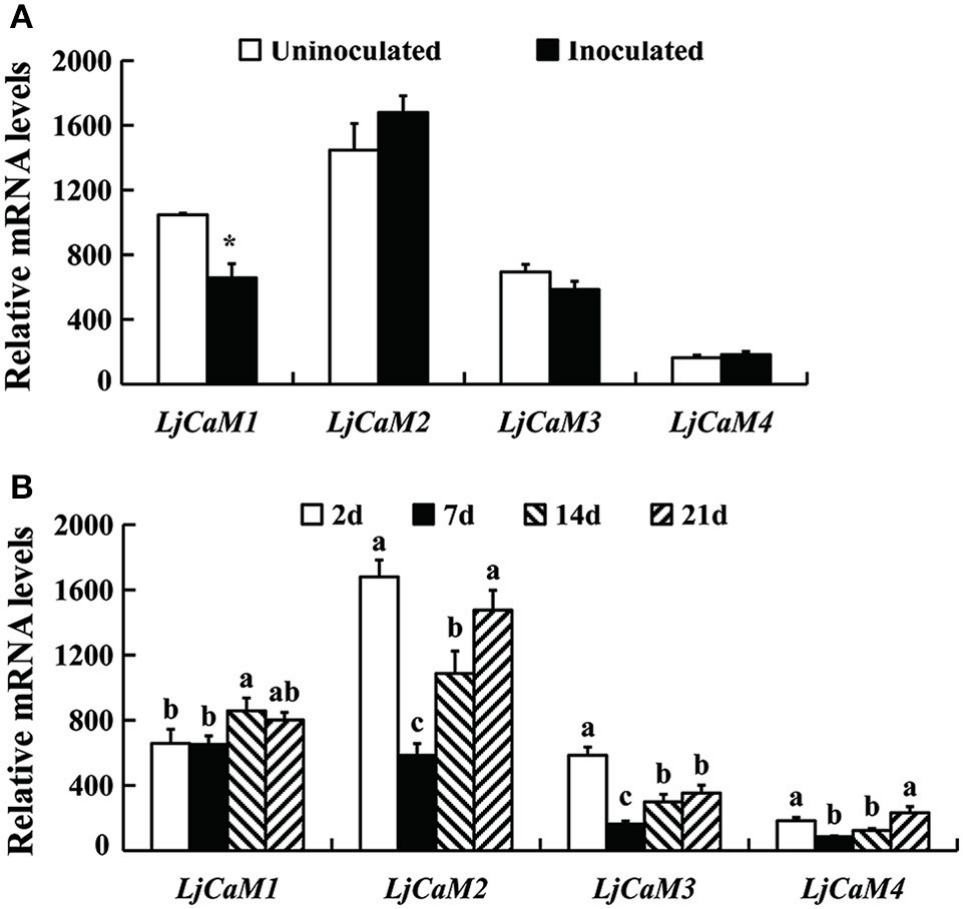
**Gene expression analysis by Q-PCR for the ***LjCaM***s genes**. Roots inoculated with *M. loti* strain NZP2355. Each sample consisted on a pool of at least 10 individual plants. Expression levels are shown relative to the housekeeping *Ubiquitin* gene. The *NIN* gene was used as a positive control to demonstrate that the nitrogen fixation symbiotic model was successfully constructed. **(A)** the relative mRNA expression of LjCaM1, LjCaM2, LjCaM3, and LjCaM4 at 2 days after inoculation comparing with that of uninoculated roots; **(B)** the relative mRNA expression of LjCaM1, LjCaM2, LjCaM3, and LjCaM4 at 2, 7, 14, and 21 days after inoculation. Asterisks indicate significantly different from control, ^*^P < 0.05; ^abc^Mean values with different small letters were *P* < 0.05.

Based on the diversity of the 3' untranscription region obtained by 3'-RACE cloning, we designed the fluorescent quantitative PCR primers (Table 1). From Figure 6, it is not difficult to find that the expression of *LjCaM*1, *LjCaM*2, *LjCaM*3, and *LjCaM*4 is significantly different at different infection stages. Among them, the relative expression of *LjCaM*2 is the highest, and that of *LjCaM*4 is the lowest. The epidermal cells were infected by rhizobia after inoculation 2 day. At this development stage, the expression of *LjCaM*1 was significantly down-regulated, which indicates that it might be involved in the early rhizobia infection epidermal cells stage (Figure 6A). When the bacteria infect the cortex cells (after inoculated 7 days), the expression of *LjCaM*2, *LjCaM*3, and *LjCaM*4 is significantly down-regulated. The expression of *LjCaM*1, *LjCaM*2, and *LjCaM*3 is significantly up-regulated after inoculated 14 days (Figure 6B).

## Discussion

### The expression of LjCaMs during rhizobial infection

A defining characteristic of plant CaMs is that a series of isoforms of CaM exist in a single plant species. The functional redundancy of genes cannot be ruled out; however, accumulating evidence suggests that multiple CaM isoforms may have distinct and significant functions (Heo et al., 1999; Karita et al., 2004; Phean-o-pas et al., 2005; Gifford et al., 2013; Abbas et al., 2014). During the gene duplication events, there are two different kinds of homology: orthology (vertical) and paralogy (horizontal) (Das et al., 2016). Orthologs are defined as genes in different species that have originated in evolution from an ancestral gene and vertical transmission homologous genes. Orthologs frequently share the same function in the newly evolved species (Das et al., 2016). Paralog is that several homologous genes were produced by double and lateral (horizontal) of the ancestor gene in the same genome (or homologous genomes). The paralogs will undergo functional divergence (Das et al., 2016). Therefore, highly conserved CaM isoforms have been reported to modulate target proteins differently (Karita et al., 2004). Additionally, the expression of some but not all of the multiple CaM isoforms in a plant tissue is induced in response to certain stimuli (McCormack and Braam, 2003). The expression patterns of each calmodulin gene in potato are different in various tissues during development (Takezawa et al., 1995). *OsCaM1-1* has been shown to be significantly increased in leaves during osmotic stress (Kawasaki et al., 2001). In tobacco, *NtCaM13* was shown to be exclusively expressed in the root; however, 13 related *CaM* genes were expressed in almost all of the examined organs (Yamakawa et al., 2001). Steady state expression levels of *CaM*s have been reported to be modulated in different special phase of plant growth (Delk et al., 2005; Yang et al., 2014) as well as in response to external stimuli (Heo et al., 1999; Phean-o-pas et al., 2005; Gifford et al., 2013; Abbas et al., 2014). Therefore, the modulation of gene expression of a CaM isoform in specific organs allows for the possibility of functioning in a timely fashion.

Calmodulin is known to involve in bacterial infection (Liao et al., 2012; Shimoda et al., 2012; Miller et al., 2013). In an effort to clarify the roles of *LjCaM*s in nitrogen fixation symbiosis of *L. japonicus*, the expression of four mRNAs of calmodulin isoforms (LjCaM1, LjCaM2, LjCaM3, and LjCaM4), in a single organ and at different nodule development stages was examined by Q-PCR. Four LjCaM isoforms are involved in different rhizobia infection stages. The maintenance of these genes suggests that they are unlikely to be fully redundant in function. GmCaM4 did not activate NAD kinase at all and has different expression pattern in contrast to SCaM-1 (Sang et al., 1995). LjCaM4 and GmCaM4 are orthologs, which indicates that they may have other uncertain functions. However, LjCaM4 and LjCaM1 are paralogs. There is no strictly definition of paralogs in function. They may be similar or different (although share a certain degree of similar structure), or even no function (such as pseudogene; Das et al., 2016). Considering that these LjCaM sequences have been conserved by natural selection, it is quite possible that these genes have significant physiological importance. Thus, competition between CaM isoforms for target proteins may exist in *L. japonicus* root nodule symbiosis.

Although *CaM* expression is ubiquitous among cell types, the protein concentrations may vary between different cell types (Poohvaiah and Reddy, 1993). Further research is required in order to determine whether the seven *L. japonicus CaM* genes have different roles or regulatory processes. The regulation of bacteria-induced calmodulin gene expression will be studied using transgenic plants carrying the promoter of the calmodulin isoform fused to the GUS reporter gene.

### Organ-specific expression of *LjCML*s

Fifty, thirty-two and fifty-two members of the CML family have been reported in Arabidopsis (McCormack and Braam, 2003), rice (Boonburapong and Buaboocha, 2007) and tomato (Munir et al., 2016), respectively. Similar to LjCaMs, these CML members are composed of EF hands without other functional domains. As shown in Figure 3, some CMLs have diversity in loop sequences compared with those of known CaMs, although most of the EF hand domains maintain the strict conservation of the Ca^2+^-binding residues (McCormack and Braam, 2003). Thus, the Ca^2+^ binding affinity of CMLs might be different from that of CaMs. There was also a difference in Ca^2+^ affinity between CMLs (Chigri et al., 2012). Through the change of cytosolic Ca^2+^ signals, CMLs bind to target proteins and alter their activities, which subsequently affects physiological responses to a great deal of target stimuli received by plant cells (McCormack and Braam, 2003; Yang and Poovaiah, 2003). The number of CMLs in land plants reflects the alterations of gene expression in response to various environmental cues (Boonburapong and Buaboocha, 2007; Park et al., 2010; Ruge et al., 2016; Zhu et al., 2016). CML4/5-like proteins play a potential role in vesicle transport within the plant endomembrane system (Ruge et al., 2016). *Arabidopsis* CML8 expression is strongly and transiently induced by *Pseudomonas syringae* and plays a key role in plant immunity against *P. Syringae* (Zhu et al., 2016). According to the Lotus gene expression atlas, some *LjCML*s genes are highly expressed in specific organs such as *LjCML1, LjCML11*, and *LjCML16* in flowers, *LjCML6, LjCML7*, and *LjCML12* in nodules, *LjCML14* in roots, and *LjCML19* in seeds. At different developmental stages of nodule formation, the expression of a few *LjCML*s is significantly different. The gene expression of these *LjCML*s needs to be further explored by some detection means such as Q-PCR, and more related studies will be carried out in the future.

## Author contributions

JqL, ZQ, YZ participated in the design of the study; JqL, JD, MS analyzed the data; CD, JL, CH assisted with bioinformatic analysis and sequences alignment; JT participated in 3′-RACE; MY, YH, RY participated in qRT-PCR and data analysis; JqL initiated and supervised the study and wrote the manuscript. All authors read and approved the final version.

### Conflict of interest statement

The authors declare that the research was conducted in the absence of any commercial or financial relationships that could be construed as a potential conflict of interest.
